# Attitudes and Perceptions of Caregivers of person with Frontotemporal Dementia in Peru: Preliminary results

**DOI:** 10.1002/alz.093076

**Published:** 2025-01-09

**Authors:** Sheila Castro‐Suarez, Maryenela Z. Illanes‐Manrique, Maria Meza‐Vega, José Cuenca, Serggio Lanata, Mario Cornejo‐Olivas

**Affiliations:** ^1^ Global Brain Health Institute (GBHI), University of California San Francisco, San Francisco, CA USA; ^2^ CBI en Demencias y Enfermedades Desmielinizantes del Sistema Nervioso, Instituto Nacional de Ciencias Neurológicas, Lima Peru; ^3^ Neurogenetics Research Center, Instituto Nacional de Ciencias Neurológicas, Lima Peru; ^4^ Neurogenetics Working Group, Universidad Científica del Sur, Lima Peru; ^5^ School of Medicine, Universidad Nacional Mayor de San Marcos, Lima Peru; ^6^ Memory and Aging Center, UCSF Weill Institute for Neurosciences, University of California, San Francisco, San Francisco, CA USA; ^7^ Global Brain Health Institute, San Francisco, CA USA; ^8^ School of Medicine, Villa El Salvador, Lima Peru

## Abstract

**Background:**

Frontotemporal dementia (FTD) is a clinical syndrome characterized by progressive changes in behavior, executive function, or language. In Latin America, persons with FTD are underrecognized or diagnosed late. There is a lack of knowledge about the experiences have on families.

**Method:**

We used a mixed methods approach consisting of qualitative, semi‐structured interviews that were recorded, transcribed, and analyzed using thematic analysis. Local IRB approval was obtained for this study.

**Result:**

We interviewed fourteen family caregivers of persons with FTD, ten of them (71.4%) were caregivers of persons with behavioral variant FTD (bvFTD). There was female predominance (78.5%). The average of education was 14.5±2,3 years. Half were sole caregivers, seven were children, five were spouses, and two were sisters. The key themes emerging from qualitative interviews were difficulties surrounding the diagnostic process, the impact of knowing the diagnosis, and challenges surrounding caregiving. Caregivers stressed how tedious it is to access health services and obtain specialized care and the lack of knowledge of the disease among health professionals. Caregivers commented on the perceived lack of skills of some professionals when informing about the diagnosis and prognosis of a neurodegenerative disease. Knowledge of FTD prognosis generates anguish, hopelessness, and frustration among caregivers. Caregivers who are sons or daughters often adopt leadership roles within the family, especially from financial and caregiving point of view. They also often change or postpone life/professional plans. On the other hand, if the caregiver is the wife or husband, the absence of their partner in the home stands out as a main stressor, even though they remain together.

**Conclusion:**

Our preliminary results highlight the importance of providing accessible, plain‐language education and support to caregivers of persons with FTD, as well as the opportunity to improve training on healthcare professionals. The impact of caring for patients with FTD appears to be dependent on the parental relationship.

